# Polyoxometalates as Effective Nano-inhibitors of Amyloid Aggregation of Pro-inflammatory S100A9 Protein Involved in Neurodegenerative Diseases

**DOI:** 10.1021/acsami.1c04163

**Published:** 2021-06-03

**Authors:** Himanshu Chaudhary, Igor A. Iashchishyn, Nina V. Romanova, Mark A. Rambaran, Greta Musteikyte, Vytautas Smirnovas, Michael Holmboe, C. André Ohlin, Željko M. Svedružić, Ludmilla A. Morozova-Roche

**Affiliations:** † Department of Medical Biochemistry and Biophysics, Umeå University, Umeå 90187, Sweden; ‡ Department of Chemistry, Umeå University, 90187 Umeå, Sweden; § Institute of Biotechnology, Life Sciences Center, Vilnius University, Vilnius LT-10257, Lithuania; ∥ Department of Biotechnology, University of Rijeka, Rijeka HR 51000, Croatia

**Keywords:** amyloid, amyloid-neuroinflammatory cascade, fibrils, inhibition, S100A9, polyoxometalate, decaniobate, titanoniobate

## Abstract

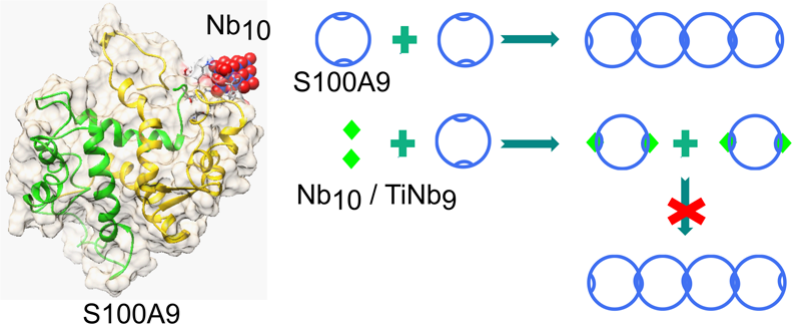

Pro-inflammatory and amyloidogenic S100A9 protein is central to the amyloid-neuroinflammatory cascade in neurodegenerative diseases. Polyoxometalates (POMs) constitute a diverse group of nanomaterials, which showed potency in amyloid inhibition. Here, we have demonstrated that two selected nanosized niobium POMs, Nb_10_ and TiNb_9_, can act as potent inhibitors of S100A9 amyloid assembly. Kinetics analysis based on ThT fluorescence experiments showed that addition of either Nb_10_ or TiNb_9_ reduces the S100A9 amyloid formation rate and amyloid quantity. Atomic force microscopy imaging demonstrated the complete absence of long S100A9 amyloid fibrils at increasing concentrations of either POM and the presence of only round-shaped and slightly elongated aggregates. Molecular dynamics simulation revealed that both Nb_10_ and TiNb_9_ bind to native S100A9 homo-dimer by forming ionic interactions with the positively charged Lys residue-rich patches on the protein surface. The acrylamide quenching of intrinsic fluorescence showed that POM binding does not perturb the Trp 88 environment. The far and near UV circular dichroism revealed no large-scale perturbation of S100A9 secondary and tertiary structures upon POM binding. These indicate that POM binding involves only local conformational changes in the binding sites. By using intrinsic and 8-anilino-1-naphthalene sulfonate fluorescence titration experiments, we found that POMs bind to S100A9 with a *K*_d_ of ca. 2.5 μM. We suggest that the region, including Lys 50 to Lys 54 and characterized by high amyloid propensity, could be the key sequences involved in S1009 amyloid self-assembly. The inhibition and complete hindering of S100A9 amyloid pathways may be used in the therapeutic applications targeting the amyloid-neuroinflammatory cascade in neurodegenerative diseases.

## Introduction

Spontaneous self-assembly of polypeptides into ordered amyloid aggregates emerged as a universal phenomenon involved in a number of human pathologies, including neurodegenerative diseases such as Alzheimer’s and Parkinson’s.^1^ Amyloid is characterized by a generic structure of cross-β-sheet in its core, and this structure is common to all amyloid fibrils formed by various structurally unrelated polypeptides. Amyloids can be deposited in various tissues and organs causing damage due to their accumulation and cytotoxicity. The latter is associated with small self-assembled entities known as amyloid oligomers.^2^ Since the neurodegenerative amyloid illnesses are concomitant to aging, the growing elderly population leads to a significant increase in the incidence of neurodegenerative conditions and, ultimately, to increasing social and health care costs. By statistical estimate, more than 150 million people worldwide will be affected by Alzheimer’s disease by 2050 and this will triple the current number.^3,4^ In spite of significant efforts to decipher the causes of the amyloid neurodegenerative diseases, up to date, there are only symptomatic treatment options available, but no cures are able to reverse amyloid formation.

Over the past decades, nanoparticles and overall nanosized materials were identified for their great potential in multifunctional therapies and treatment modalities in amyloid-related diseases.^5−8^ It was demonstrated that depending on their physicochemical characteristics, such as size, charge, shape, and composition, nanomaterials can significantly affect the protein amyloid fibrillation and therefore may be used for amyloid inhibition, reversal, and also detection. Among nanomaterials, polyoxometalates (POMs) represent a large, diverse, and remarkably alterable class of inorganic compounds with promising biological activities.^9^ POMs are polyoxyanions of transition metals from the groups five (V, Nb, and Ta) and six (Mo and W), usually present in their highest oxidation state. The sizes and three-dimensional structural motifs of many POMs are similar to the water-solubilized fullerene derivatives. Recently, they were reported to possess amyloid-inhibiting properties and anti-HIV-1P activity.^10,11^ The properties of POMs can be also tuned with respect to their elemental composition, structure, charge density, redox potential, acidity, and solubility, depending on the specific applications. Since POMs are discrete molecules with high symmetry, their structures are often known with high confidence. While research into POMs was historically focused on V, W, and Mo POMs, in the past decade, there was a rapid growth in the synthesis and applications of Nb and Ti POMs.^12^ Advantages of these latter classes of POMs include resistance to oxidation and reduction, lower tendencies to speciation, and better stability at neutral and elevated pH. These properties make them suitable for biological applications, as there is reduced likelihood of spontaneously formed interfering species.

**Figure 1:**
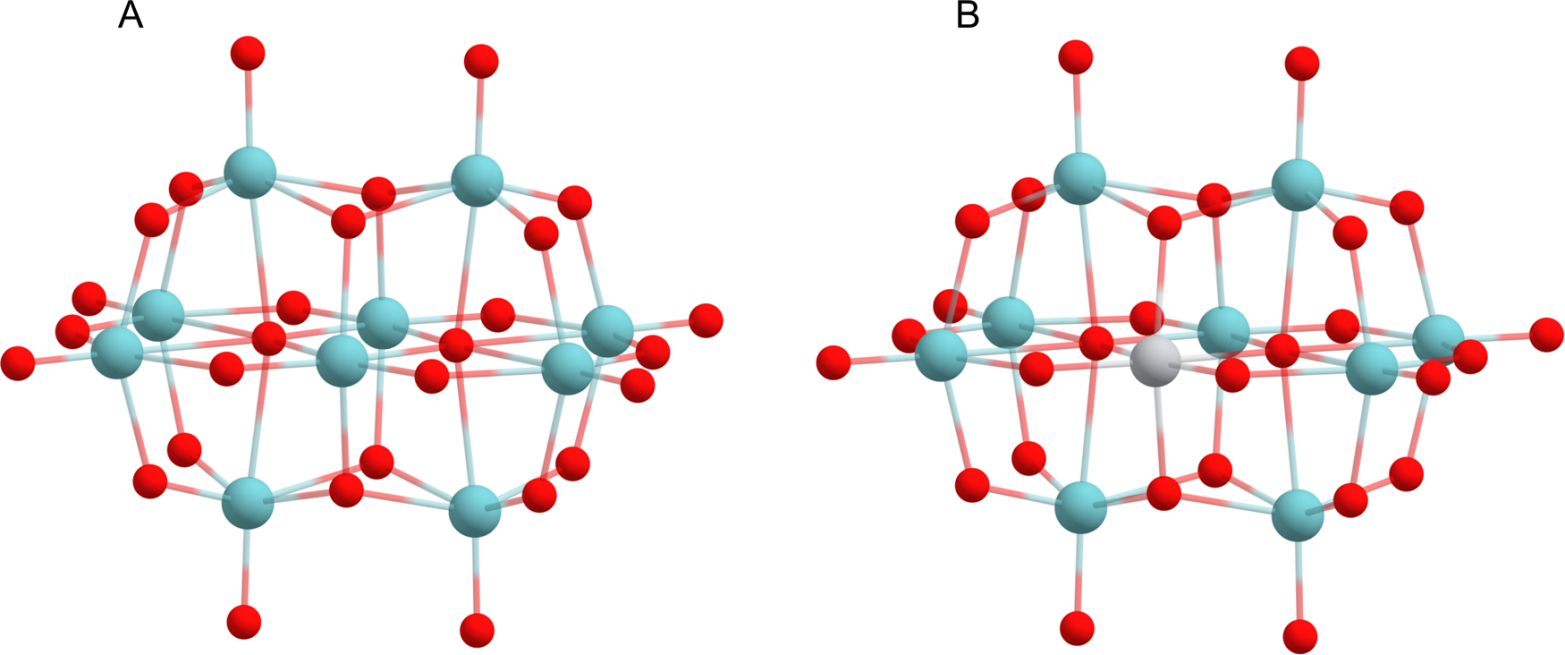
Structures of (A) decaniobate Nb_10_ = [Nb_10_O_28_]^6–^ and (B) monotitanoniobate TiNb_9_ = [TiNb_9_O_28_]^7–^. Nb atoms are shown by blue spheres, Ti atom by a gray sphere, and oxygen atoms by red balls.

Here, we have demonstrated that two polyoxoniobates, *viz.*, decaniobate [N(CH_3_)_4_]_6_[Nb_10_O_28_] (Nb_10_)^13,14^ and monotitanoniobate [N(CH_3_)_4_]_7_[TiNb_9_O_28_] (TiNb_9_)^15^ (Figure 1), can effectively hinder amyloid formation of pro-inflammatory protein S100A9. As a pro-inflammatory molecule or alarmin, S100A9 is involved in the inflammatory signaling pathways and found to be abundant in cancers and many ailments associated with inflammatory processes.^16−18^ It has been shown that S100A9 is involved also in Alzheimer’s disease,^19,20^ traumatic brain injuiry,^21,22^ Parkinson’s disease,^23^ malaria,^24^ cerebral ischemia,^25^ obesity,^26^ and cardiovascular disease.^27^ The abundance of S100A9 mRNA was found to be linked to aging in various mammalian tissues, including the central nervous system, indicating that S100A9 is involved in the age-dependent inflammation.^28^ Recently, we have found that in contrast to other pro-inflammatory molecules, S100A9 can easily self-assemble into amyloids,^29^ which may lead to losing its signaling functions and to acquiring a new functionality such as amyloid cytotoxicity. Indeed, S100A9 amyloids were found to be cytotoxic and even exceed the toxicity of amyloid-β (Aβ) peptide amyloids in Alzheimer’s disease^29^ and α-synuclein amyloids in Parkinson’s disease.^23^ Therefore, the rising S100A9 level sustained during inflammation may lead to its amyloid formation and deposition, as we have shown to occur during Alzheimer’s^29^ and Parkinson’s diseases,^23^ traumatic brain injury,^22^ and in the aging prostate.^30^ By contrast, in an Alzheimer’s disease mouse model, the knockdown of S100A9 produced a significant reduction in the amount of amyloid plaques and attenuated memory impairment.^31^ Moreover, S100A9 is prone to co-aggregate with Aβ peptide and α-synuclein, which may further exacerbate the amyloid-neuroinflammatory cascade in the corresponding neurodegenerative diseases.^23,32^ Importantly, the cerebrospinal fluid levels of S100A9 closely match those of Aβ peptide in Alzheimer’s disease, vascular dementia, and mild cognitive impairment,^33^ further emphasizing the direct involvement of S100A9 together with Aβ peptide in the amyloid-neuroinflammatory cascade in these diseases.

It became increasingly evident that the neurodegenerative diseases are characterized by a long preclinical or silent phase when the pathological processes are initiated but not yet manifested in clinical symptoms. The inflammation could be a leading course of preclinical pathology as we have shown that the S100A9-driven amyloid-neuroinflammatory cascade and sustained inflammation in traumatic brain injury may indeed lead to Alzheimer’s disease development.^22^ Therefore, if the amyloid accumulation of S100A9 can be finely tuned and inhibited, which we demonstrate in the present research, it may open a possibility to halt the whole amyloid-neuroinflammatory cascade and aggregation of other proteins involved in it at the preclinical stages and prevent the development of neurodegeneration. Here, by using a range of spectroscopic techniques, atomic force microscopy (AFM) imaging, and molecular dynamics (MD) simulation, we have explored the properties of two POMs, specifically, Nb_10_ and TiNb_9_, in their capacity to bind to S100A9 and inhibit its amyloid fibrillation, which bears prospective therapeutic significance.

## Materials and Methods

### Synthesis and Characterization of [N(CH_3_)_4_]_6_[Nb_10_O_28_]·2H_2_O (Nb_10_) and [N(CH_3_)_4_]_7_[TiNb_9_O_28_]·3H_2_O (TiNb_9_)

Nb_10_ (*M*_w_, 1896 Da) and TiNb_9_ (*M*_w_, 1851 Da) were synthesized based on published methods.^13,34^ In the general procedure, Nb_10_ was synthesized by microwave irradiation (400 W) of a 5 mL sealed glass vial containing a suspension of Nb_2_O_5_·*n*H_2_O (1.0 g, 3.1 mmol, 18% H_2_O w/w) in an aqueous solution of [N(CH_3_)_4_][OH]·5H_2_O (TMAOH; 0.67 g, 8.24 mmol, 2 mL) at 180 °C for 15 min, yielding autogenic pressures of 12–14 bars. TiNb_9_ was synthesized by irradiation of a suspension of Nb_2_O_5_·*n*H_2_O (3.0 g, 9.3 mmol, 18% H_2_O w/w) and TiO_2_ (0.52 g, 6.49 mmol) in an aqueous solution of TMAOH (2.5 g, 13.8 mmol, 10 mL) at 180 °C in a 20 mL sealed glass vial for 30 min. Following microwave irradiation and cooling, the suspensions were gravity-filtered through paper (No 2), the liquors were kept, and the products were precipitated with acetone (Nb_10_) or 2-propanol/acetonitrile (TiNb_9_). The precipitated Nb_10_ was filtered on a fritted filter (M) under suction and then oven-dried at 90 °C. The precipitated TiNb_9_ was extracted into methanol and filtered through a 0.20 μm PTFE syringe filter to remove any remaining solids, prior to drying *in vacuo*. The identity and purity of Nb_10_ and TiNb_9_ were confirmed by using Raman and ^17^O NMR spectroscopy, and the spectra were in agreement with the literature data.^34,35^

### Amyloid Fibril Formation

S100A9 was expressed in *Escherichia coli* and purified as described previously.^36^ Lyophilized S100A9 was dissolved on ice in 50 mM HEPES, pH 7.0. Before incubation, S100A9 samples were filtered through a 0.22 μm spin membrane filter to remove any preformed aggregates. The amyloid formation was carried out by incubating S100A9 in 50 mM HEPES, pH 7.0 and 42 °C. We used the following molar ratios of S100A9 to the corresponding POM during amyloid incubation: 1:0.5, 1:1, 1:2; 1:4, 1:8, and 1:10. No precipitation of POMs was noticed in our experiments.

### Thioflavin-T (ThT) Fluorescence Assay

ThT dye, binding specifically to the amyloid cross-β-sheet structures, enables monitoring the kinetics of amyloid self-assembly. A ThT fluorescence kinetic assay was performed as described previously.^37^ The samples of 75 μM S100A9 were transferred into Corning 96 black well plates with transparent bottoms, and then 20 μM ThT was added to each well. The sample volume was kept at 200 μL per well. The plates were immediately covered, placed into a Tecan F200 PRO plate reader, and incubated at 42 °C for 150 h. ThT fluorescence was recorded each 10 min by using 432 rpm orbital shaking. Filters at 430 and 495 nm wavelengths with a 20 nm band width each were used for excitation and emission, respectively. Each protein sample was incubated in triplicate.

### Intrinsic Fluorescence and Quenching Experiments

The samples of 4 μM native S100A9 were titrated with Nb_10_ and TiNb_9_, respectively, in a 2 mm quartz cuvette using 50 mM HEPES, pH 7.0 and at room temperature. Intrinsic fluorescence spectra were acquired by using a Jasco spectrofluorometer FP 6500. Excitation at 295 nm and emission between 300 and 450 nm were used with the excitation and emission slits set at 5 nm. The spectra were averaged over three scans recorded at 200 nm/min. The maximum positions of fluorescence spectra of S100A9, containing single Trp 88 residue, were determined by using a first derivative method. Fluorescence quenching by acrylamide was performed for S100A9 alone and at 1:1 molar ratios of S100A9 to POMs under the same conditions. The quenching sphere of action model was used to fit the acrylamide fluorescence titration curves as reported previously.^38^

### Nb_10_ and TiNb_9_ Titration of Preformed S100A9 Amyloid Fibrils Monitored by ThT Fluorescence

Initially, 150 μM S100A9 was aggregated in 50 mM HEPES, pH 7.0 and 42 °C for 48 h. ThT (20 μM) was added to S100A9 (75 μM) solutions containing mature fibrils, which were subsequently titrated with Nb_10_ and TiNb_9_, respectively. ThT fluorescence spectra during titration were recorded in a 2 mm quartz cuvette at room temperature by using a Jasco fluorometer FP 6500. The excitation wavelength was set at 450 nm, emission spectra were recorded between 475 and 550 nm, and the slit width was set at 10 nm for both excitation and emission. The spectra were averaged over three repeats recorded at 200 nm/min.

### 8-Anilino-1-Naphthalene Sulfonate (ANS) Fluorescence of S100A9 in the Presence of Nb_10_ and TiNb_9_

The binding of POMs by native and amyloid S100A9 was monitored by using fluorescence dye ANS. The S100A9 aggregation was performed in the presence of different concentrations of Nb_10_ and TiNb_9_ at 42 °C under quiescent conditions and the aliquots were collected after 0, 12, and 150 h incubation. ANS (120 μM) was added to each sample and its fluorescence was measured using a Jasco spectrofluorometer FP 6500. Excitation at 365 nm and emission between 380 and 650 nm were used with the excitation and emission slits set at 5 nm. The spectra were averaged over three scans recorded at 200 nm/min.

### Kinetic Curve Fitting

Kinetic traces of S100A9 amyloid formation demonstrate the hyperbolic dependence, which is indicative of the isodesmic polymerization.^39,40^ The extent of reaction (fraction of the total number of reacted groups) is described by the following equation:^41^
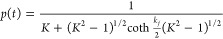
1where ; *k_f_* and *k_b_* are the forward and backward reaction rates for an individual step.

The limit of eq 1 when the backward reaction is negligible becomes^41^
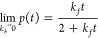
2

In the present work, depolymerization rates were not measured and assumed that depolymerization is negligible, and therefore, eq 2 was used to fit the kinetic traces and to extract the effect of POMs on the rate of S100A9 amyloid formation.

The dependence of kinetics rates on concentration of POMs has an inverse hyperbolic character, and therefore the concentration dependence of the fitted rates was modeled using a simple exponential function:
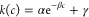
3where β is the parameter illustrating relative susceptibility of the rate constant to the amount of POMs, while α and γ are the fitting constants.

### Fitting of Fluorescence Titration Curves

Fitting of the titration curves of POM binding to S100A9 monitored by ANS dye and intrinsic Trp 88 fluorescence was performed by using the ligand-receptor binding equation and assuming one type of binding site on the surface of native or amyloid S100A9 complexes
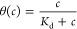
4where θ(*c*) is the fraction of the POM-S100A9 complex, *c* is the POM concentration, and *K*_d_ [μM] is the dissociation constant.

### AFM Imaging

AFM imaging was performed by using a BioScope Catalyst AFM (Bruker), operating in peak force mode in air. The scan rate was 0.51 Hz, and resolution was 512 × 512 pixels. Bruker MSLN and SLN cantilevers were used in all measurements. Imaging was also conducted using a PicoPlus AFM (Molecular Imaging), equipped with a 100 × 100 μm scanner, operating in tapping mode in air. The resonance frequency was set in a 170–190 kHz range, scan rate at 1 Hz, and resolution at 512 × 512 pixels. For ambient imaging, 20 μL of each sample was deposited on the surface of freshly cleaved mica, kept for 30 min, washed five times with 200 μL of deionized water, and left dry overnight at room temperature. Heights of amyloid fibrils were measured in the AFM cross sections by using Bruker Nanoscope analysis software.

### Circular Dichroism (CD) Measurements

CD spectra were recorded by using a Jasco J-810 spectropolarimeter. The far UV CD measurements were performed in a quartz cuvette with a 1 mm spectral pathway using a scan speed of 50 nm/min and band width at 1 nm and with five repeats. The near UV CD spectra were recorded using a 10 mm spectral pathway, with a scan speed of 50 nm/min, a band width of 1 nm, and five repeats. The optical absorbances of protein samples over buffer were in a range of 0.4–1. The CD spectra in the corresponding UV region of buffer and POMs were subtracted from the S100A9-POM spectra. Weighted spectral differences (WSD) between the corresponding pairs of spectra of S100A9 with POMs *versus* S100A9 alone were calculated as described previously.^42^ Standard deviations of WSD were calculated from five repeats of individual spectra.

### MD Simulation: All Electric Field Maps

All electric field maps were calculated using the Adaptive Poisson–Boltzmann Solver (APBS) approach.^43^ For MD simulation, we used the S100A9 structure averaged over 10 NMR structural conformers presented in the original PDB file 5I8N,^44^ by applying the NMR structure averaging tool in UCSF Chimera.^45^ Ten NMR conformers corresponded to a 0.1–10 ns time frame,^44^ while in MD simulation, we have achieved a significantly more detailed picture of molecular changes over a 100 ns time period, including 1000 different snapshots. PDB formats for S100A9^44^ were converted to PQR formats using the PDB2PQR and PEOEPB force field with a PROPKA set at pH 7.2. S100A9 molecules from PDB files were protonated using GAFF fields. Potential maps were calculated in aqueous 150 mM NaCl solutions and 37 °C using single Debye–Hückel boundary conditions.

### Force Field Parameterization of Nb_10_ and TiNb_9_

Geometries of the Nb_10_ and TiNb_9_ clusters were optimized by density functional theory (DFT), using the Gaussian software (G16 rev. A.03) and an implicit polarizable continuum model^46^ at the PBE0^47^/def2-tzvp^48^ level of theory. Partial charges of all atoms in the clusters were computed as natural atomic charges by Natural Bond Order analysis.^49^ The Lennard-Jones parameters (epsilon and sigma) were subsequently optimized from Nb_10_ and TiNb_9_ clusters solvated in 600 Tip3p H_2_O molecules with charge balancing Na^+^ ions. This was accomplished by iterative MD runs controlled by a nonlinear least-squares trial-error algorithm. As a constraint, the epsilon and sigma values for all oxygen atom types were set to fall within ±10% of the water-oxygen parameters in the Tip3p H_2_O model. To further assure excellent agreement between the MD and DFT geometries, an angle restraint was used between all metal-oxygen atoms in the POMs. These optimized geometries were used further for the MD simulation of POM-S100A9 complex formation.

### All-Atom MD Calculations

The GROMACS 5.1.4 program package was used as previously described.^50^ Protein PDB coordinates^44^ were processed with pdb2gmx using the Amber99SB force field. S100A9 homo-dimer and one POM ligand were subjected to the MD simulation in each respective case. The subunit, which does not bind the POM ligand, was used as a reference state in the root-mean-square displacement (RMSD) calculations. The root-mean-square fluctuations (RMSF) were calculated by using Bio3D software. A cubic solvent box (15 nm) was used with a Tip3p model for H_2_O molecules with either 20 mM or 150 mM NaCl and with additional ions, which were required for charge neutralization. The prepared system was minimized using a combination of the steepest descent and conjugate gradient algorithms. When the most stable state was achieved, the temperature was introduced and the system was equilibrated to 310 K (NVT equilibration, V-rescale). The pressure was equilibrated to 1 atm (NPT equilibration, Parrinello–Rahman). No restraints were used for the protein or the ligand, when the system was minimized, and in NPT and NVT equilibrations.

Typical 150 ns simulations included about 450 thousand atoms and 150 million steps of numerical integration with 1 fs per step; for comparison, NMR structures give insights at the 0.1–10 ns time scale.^44^ Large simulation boxes of 20 nm each side were used, which support free diffusion of potentially interacting molecules and prevent attractive or repulsive forces created by the periodic boundary conditions. Different initial simulation setups have been explored to analyze the expected pseudo-equilibrium distribution. Following the simulations, the number of binding interactions was calculated using the built-in GROMAC functions. The results from MD calculations were analyzed using the VMD tools^51^ and Bio3D package for statistical language and program R.^52^

## Results and Discussion

### Inhibition of S100A9 Amyloid Aggregation by POMs Monitored by ThT Fluorescence

**Figure 2:**
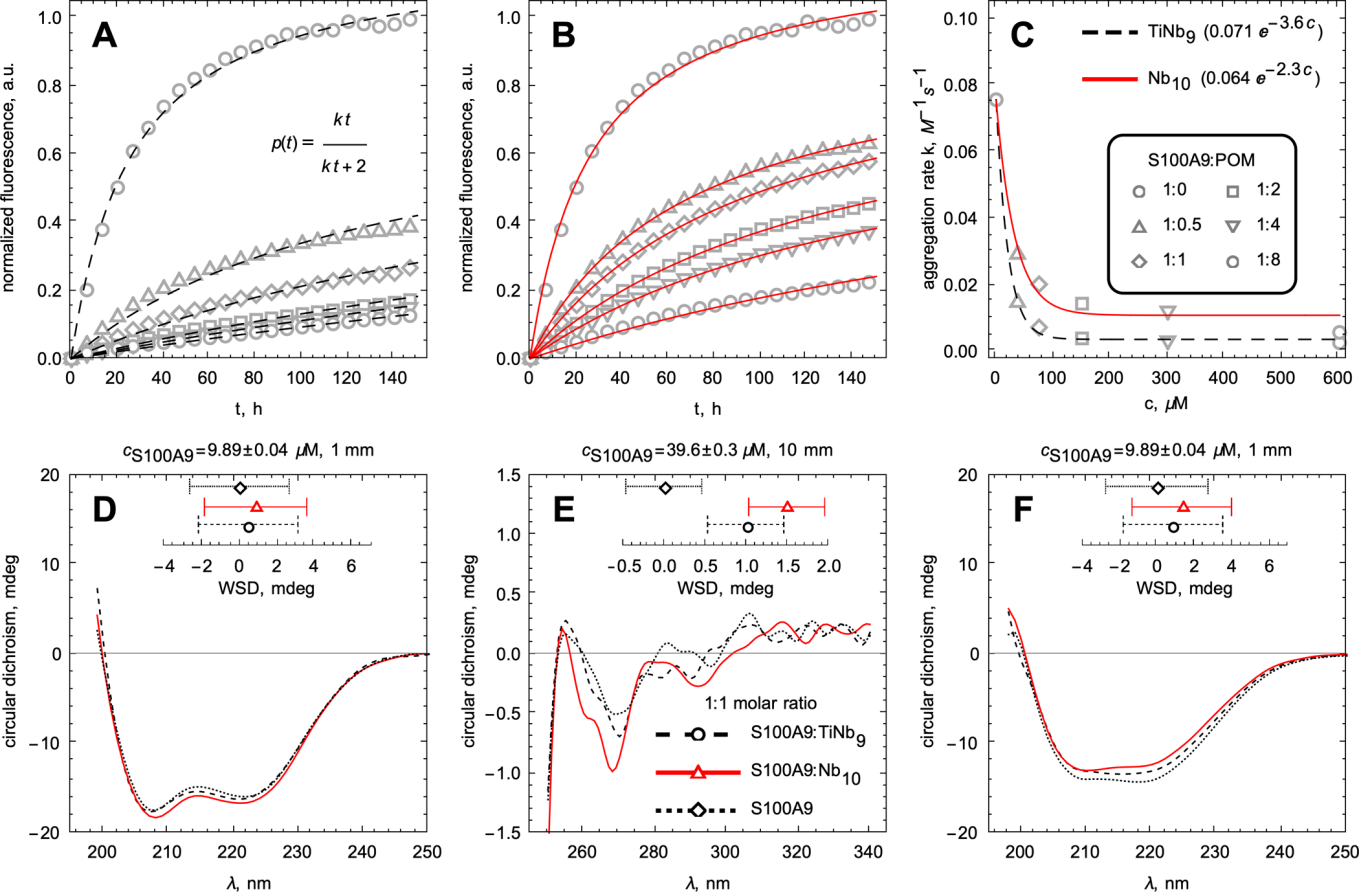
Kinetics of S100A9 amyloid formation in the presence of TiNb_9_ and Nb_10_ monitored by ThT fluorescence. (A, B) Kinetic dependencies of S100A9 amyloid formation in the presence of increasing concentrations of TiNb_9_ and Nb_10_, respectively. In both figures, the amyloid kinetics were normalized to the maximal intensity of S100A9 amyloids incubated alone. Curves in (A, B) indicate the time dependence fittings by an isodesmic polymerization model, as shown in (A, inset). (C) Concentration dependencies of polymerization rate constants (*k*) in the presence of TiNb_9_ and Nb_10_, respectively. Symbols correspond to kinetic rate constants and curves to exponential fits (explicit fitting parameters are shown in the inset to (C)). Symbols encoding the data points for specific molar ratios of S100A9 to POMs in (A–C) are indicated in the inset to (C). 75 μM S100A9, 50 mM HEPES, pH 7.0, and 42 °C. (D) Far UV CD and (E) near UV CD spectra of native S100A9 alone and with corresponding TiNb_9_ or Nb_10_. (F) Far UV CD spectra of S100A9 fibrillated alone and with corresponding TiNb_9_ or Nb_10_ for 96 h at 42 °C. The color coding of CD spectra in (D–F) is shown in the inset to (E). Molar ratios of S100A9 to the corresponding POMs are 1:1. The concentrations of S100A9 and pathlengths in CD measurements are indicated above the (D–F) panels. Weighted spectral differences are shown at the top in each of the (D–F) panels, with the error bars indicating 2 standard deviations. Their color and line codings are the same as those for the corresponding CD spectra. 10 mM PBS, pH 7.0, and 20 °C.

The kinetics of S100A9 amyloid formation in the presence of increasing concentrations of POMs were monitored by the ThT fluorescence assay as described previously,^37^ and the results are shown in Figure 2A,B. S100A9 alone self-assembles into amyloids by the nucleation-dependent polymerization mechanism,^53,54^ and its kinetics are characterized by the lack of significant lag-phase and by steep growth phase, prior to reaching the plateau level. Upon increasing POM concentrations, the rate of S100A9 amyloid self-assembly decreased as shown in Figure 2C as well as the corresponding plateau levels, reflecting the decrease in the overall amount of self-assembled amyloids (Figure 2A,B). The increasing concentrations of Nb_10_ or TiNb_9_ alone incubated with ThT under the same conditions were characterized by an order of magnitude lower ThT signal (data not shown) and therefore it was not counted in the overall ThT signal in the corresponding mixtures.

### CD Spectra in the Far and Near UV Regions of S100A9 Complexes with POMs

The interactions of POMs with S100A9 were examined by CD in the far and near UV regions, as shown in Figure 2D, E. The far and near UV CD spectra of S100A9 alone were compared with those recorded for the complexes with the molar ratio of S100A9 to Nb_10_/TiNb_9_ of 1:1. The far UV CD spectra, characterizing the secondary structure of S100A9, remained largely unchanged in the presence of corresponding POMs and displayed the shape characteristic for α-helical protein (Figure 2D). Weighted spectral differences between the corresponding pairs of the spectra of S100A9 with POMs *versus* those of S100A9 alone were calculated and also indicate that there are no significant changes of the spectra within the experimental error (Figure 2D). The near UV CD spectra of S100A9 alone and S100A9 in the complexes with corresponding POMs were characterized generally by low ellipticity and displayed no differences between all samples. These were confirmed also by weighted spectral differences (Figure 2E). This indicates that both POMs induce perturbation in the S100A9 tertiary structure on the large scale detectable by CD.

The far UV CD spectrum of S100A9 fibrillated alone was characterized by a change in the spectral shape compared to the native protein; the two distinct minima at 224 and 208 nm, characteristic for the native α-helical structure, are not clearly visible and a broad minimum at 216 nm emerges, indicating the β-sheet formation (Figure 2F). However, this spectrum is not typical of only β-sheet conformation and the contribution of α-helices and turns are still present and superimposed in the spectrum, as we have shown previously by FTIR measurements.^53,55^ Relatively low content of β-sheet in S100A9 filaments is also consistent with their short persistent length.^32,53,55^ The far UV CD spectra of S100A9 incubated with corresponding POMs at 1:1 molar ratios are characterized by some spectral perturbations relative to S100A9 fibrillated alone, but they are not significant (Figure 2F). Since upon amyloid formation, the far UV CD spectra represent the contribution from the complex ensemble of the fibrillated and nonfibrillated S100A9 conformations and each fraction bears the contribution of various secondary structures, these complex differences cannot be distinguished by far UV CD.^55^

### AFM Imaging of S100A9 Amyloids

**Figure 3:**
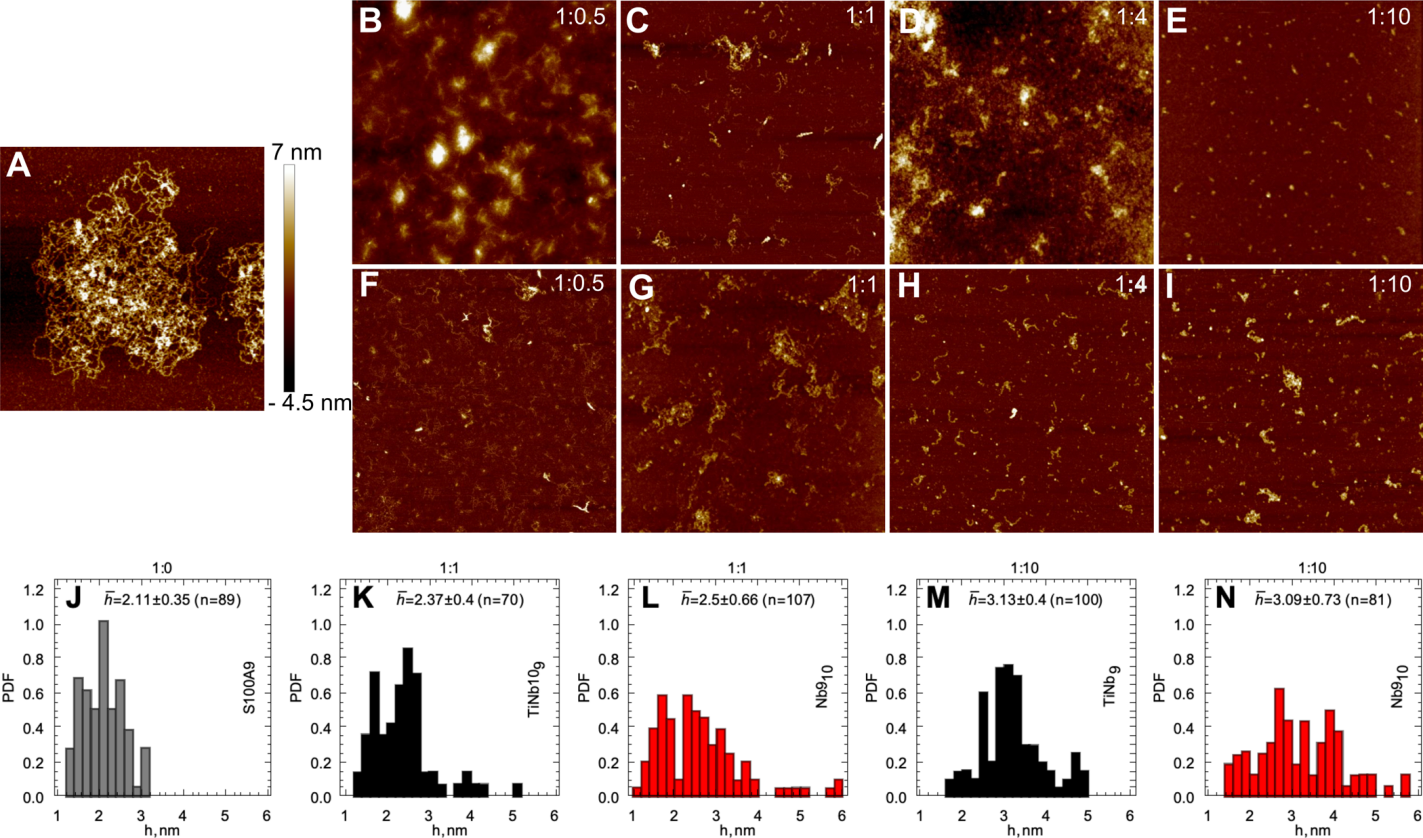
Inhibition of S100A9 amyloid formation by POMs observed by AFM imaging. (A) AFM image of S100A9 amyloid fibrils without POMs. (B–E) and (F–I) AFM images of S100A9 amyloids in the presence of increasing concentration of Nb_10_ and TiNb_9_, respectively. Molar ratios of S100A9 to corresponding POM are indicated in the figures. (J) Distribution of the AFM cross-sectional heights of S100A9 fibrils incubated alone (shown in gray). Distributions of the AFM cross-sectional heights of S100A9 aggregates formed in the presence of POMs: (K, L) at a 1:1 ratio and (M, N) at a 1:10 ratio of S100A9 to TiNb_9_ (shown in black) and Nb_10_ (shown in red) compounds, respectively. Median (*h̅*), median deviation, and number of measurements (*n*) are shown in the corresponding insets. All samples were incubated for 150 h. 75 μM S100A9, 50 mM HEPES, pH 7.0, and 42 °C. Image sizes are 2.5 × 2.5 μm in (A) and 5 × 5 μm in (B–I). The *z*-scale in AFM images is indicated by a bar with color gradient from dark brown to light yellow.

In order to monitor the changes in amyloid morphology, AFM imaging was performed on S100A9 samples in the absence and presence of POMs after 150 h incubation (Figure 3). S100A9 alone formed long, flexible fibrils with a 2.1 ± 0.35 nm median height in the AFM cross-sections and up to a micron length, which tend to intertwine into large tangles (Figure 3A,J). Remarkably, even at the 1:0.5 molar ratio of S100A9 to either of POMs, only short protofilaments were developed as monitored in the AFM images, while the long fibrils were not observed at all (Figure 3B,F). The same tendency was detected upon incubation of S100A9 at the 1:1, 1:4, and 1:10 molar ratios to corresponding POM (Figure 3). These short aggregates displayed broader distributions of the AFM cross-sectional heights with median of 2.5 ± 0.66 nm at the molar ratio of 1:10 of S100A9 to TiNb_9_ and 3.09 ± 0.73 nm of S100A9 to Nb_10_, respectively.

**Figure 4:**
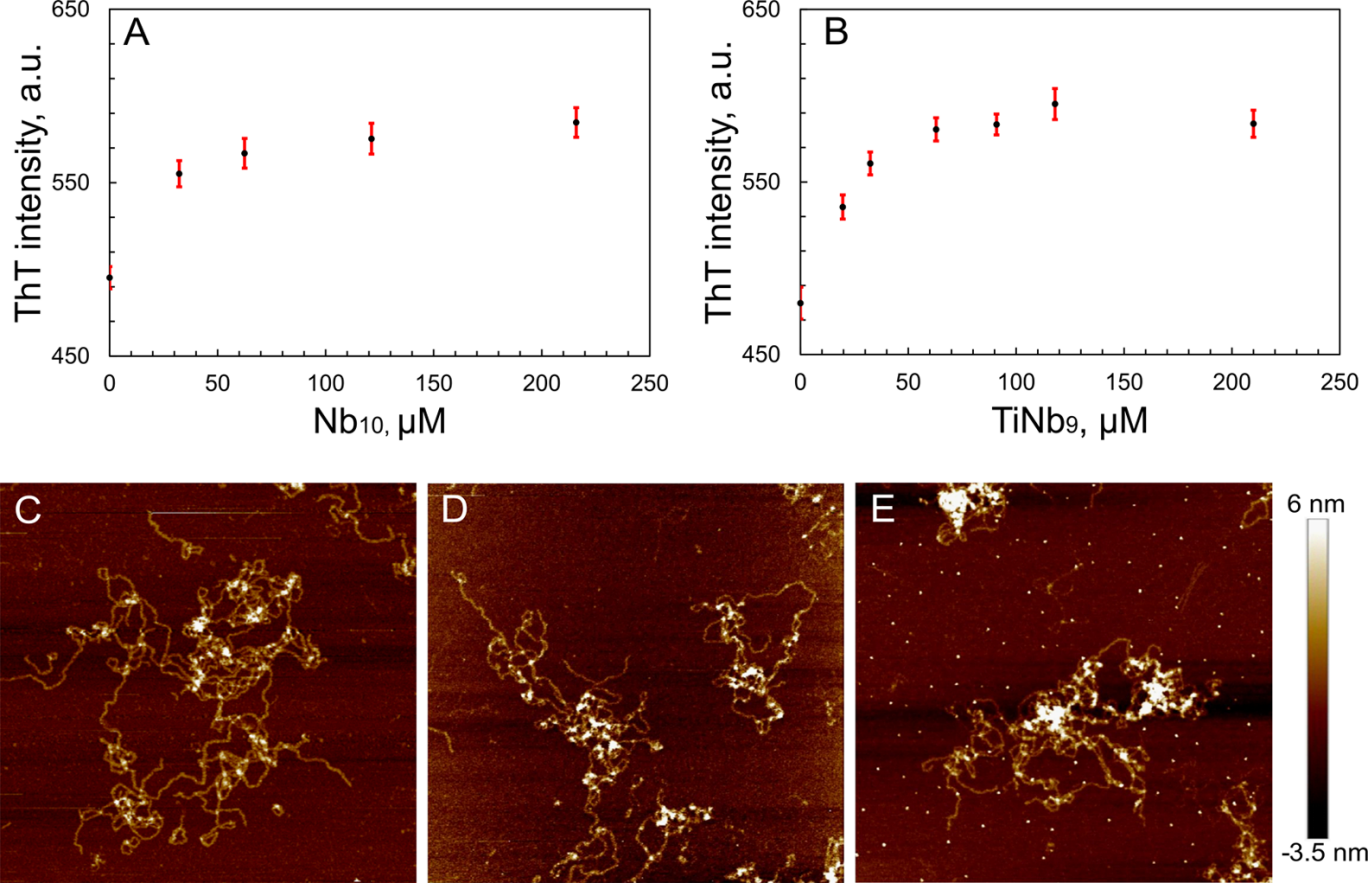
POMs do not disaggregate preformed S100A9 fibrils. (A, B) Changes in ThT fluorescence bound to S100A9 fibrils upon addition of Nb_10_ and TiNb_9_, respectively. Amyloids were preformed during 48 h incubation. (C) AFM image of S100A9 amyloids without POMs. (D, E) AFM images of S100A9 amyloids after addition of 210 μM Nb_10_ and TiNb_9_, respectively. 75 μM S100A9, 50 mM HEPES, pH 7.0, and 42 °C. Image sizes are 2.5 × 2.5 μm. The *z*-scale in AFM images is indicated on the right by a bar with color gradient from dark brown to light yellow.

Interestingly, when POMs were added to preformed amyloids of S100A9, neither a decrease in ThT signal was observed, which would be characteristic of amyloid disaggregation, nor disaggregation of long and flexible amyloid fibrils of S100A9 observed in AFM images (Figure 4). This indicates that POMs interact with native S100A9 and inhibit its amyloid formation only at the earlier stages of the self-assembly process.

### MD Analysis of the Interactions between Native S100A9 and Nb_10_ or TiNb_9_

**Figure 5:**
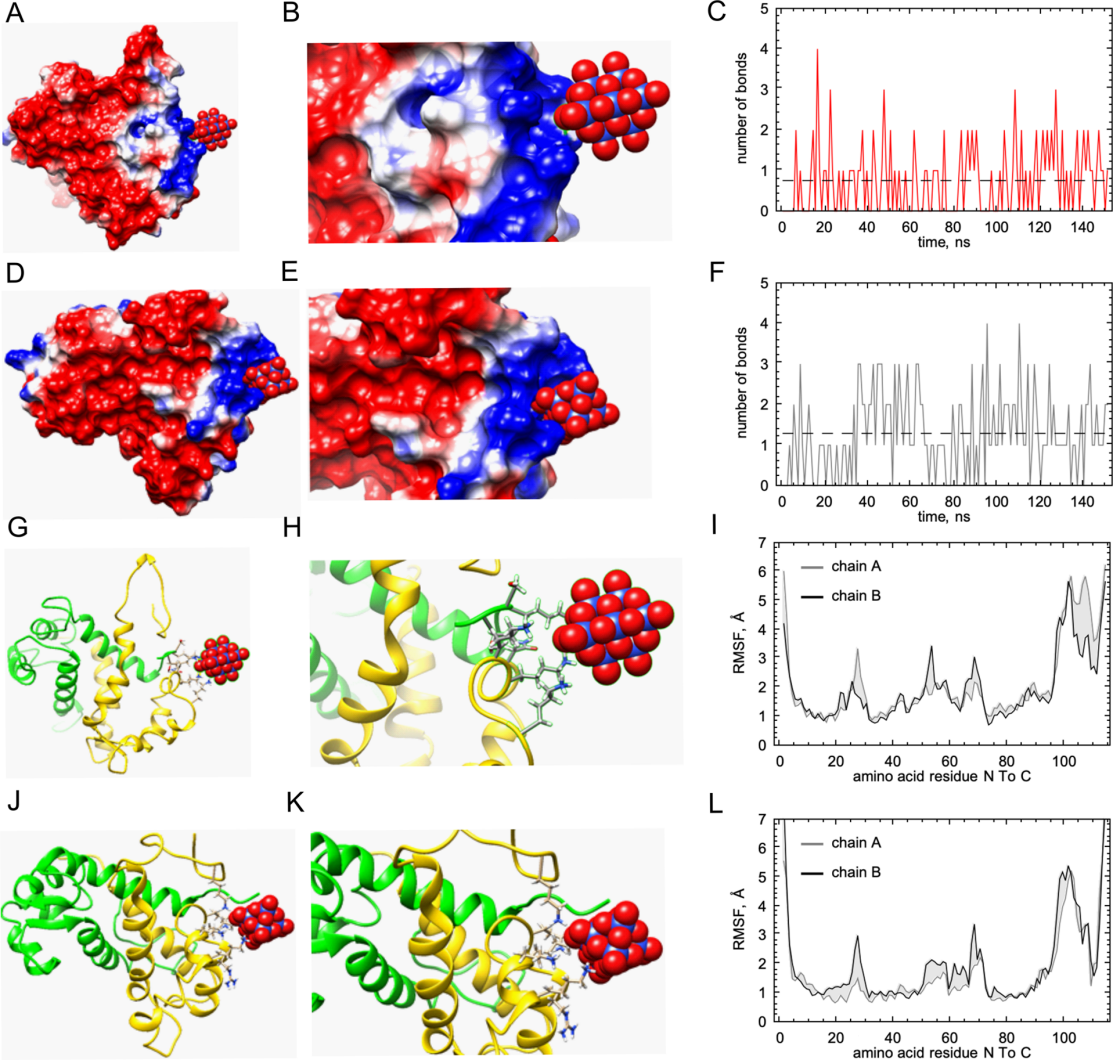
MD simulation of interactions of S100A9 with POMs. (A, D) Binding of Nb_10_ and TiNb_9_, respectively, to a subunit of S100A9 dimer. The electrostatic potentials on the protein surface are colored by a red–white–blue gradient with the values spanning from −5.0 to 5.0 kT/e. Nb_10_ and TiNb_9_ are shown as vdW spheres, where oxygen is presented in red, covering Nb atoms shown in blue and Ti in pink. (B, E) Magnified views of the complex formation of Nb_10_ and TiNb_9_, respectively, on the S100A9 surface based on ionic interactions. (C, F) Time dependence of the number of binding interactions of Nb_10_ and TiNb_9_ with S100A9, respectively. (G, J) The structure of S100A9 homo-dimer is shown in ribbons. S100A9 monomers, arbitrary denoted as chains A and B, are shown in yellow and green colors, respectively. Side chains of residues in the corresponding Nb_10_ or TiNb_9_ binding sites are shown in balls-and-sticks. (H) Nb_10_ (charge, −6) forms binding interactions with Lys50, Lys51, and Lys54 on the chain A and Lys4 on the chain B. (K) TiNb_9_ (charge, −7) forms binding interactions with Lys50, Lys51, Lys54, and Lys 106 on the chain A and no contacts with the chain B. (I, L) RMSF values for every amino acid in the chain A as shown in gray and on the chain B in black for the S100A9 complexes with Nb_10_ and TiNb_9_, respectively. Both chains A and B in the S100A9 complex with corresponding POM are characterized by identical RMSF values within statistical errors, except for the POM binding site (residues 50–55), the EF-hand Ca^2+^ binding sites (residues 20–30 and 62–70), and flexible C-terminal part of each monomer.^57^ MD was performed in the presence of 20 mM NaCl.

In order to evaluate the binding sites of POMs with S100A9, we performed MD analysis. APBS^43^ and all-atom MD calculations^50^ were carried out for S100A9-Nb_10_ and S100A9-TiNb_9_ complexes, respectively. S100A9 homo-dimer is characterized by a net charge of −12 [5I8N],^44^ while Nb_10_ and TiNb_9_ are characterized by −6 and −7 net charges, respectively, at neutral pH. The APBS analysis illustrates here the electric fields controlling the interactions between the highly charged molecules.^43^ The APBS calculations showed that S100A9 projects the dominant-negative electric potential in the surrounding space with the distinct area of concentrated positive electric field (Figure 5A,D and Figure S1). MD calculations showed that the negative electric field from S100A9 can repel the negatively charged Nb_10_ and TiNb_9_ until the ligands meet the positive patch on the S100A9 surface. The complex is observed only when Nb_10_ or TiNb_9_ molecules are captured in the narrow space, which is dominated by a positive electric field and includes Lys50, Lys51, and Lys54 (Figure 5B,E,G–K and Figure S1). This is a highly dynamic part of the protein structure,^44^ characterized by high RMSF values (Figure 5I,L), which can lead to additional ligand interactions with other amino acid side chains in its special proximity. Specifically, in the S100A9-Nb_10_ complex, Nb_10_ forms interactions with Lys50, Lys51, and Lys54 on the chain B and with Lys4 on the chain A of S100A9 homo-dimer (Figure 5C,G,H). On the other hand, in the S100A9-TiNb_9_ complex, TiNb_9_ forms interactions with Lys50, Lys51, Lys54, and Lys106 on the same chain B of S100A9 homo-dimer (Figure 5F,J,K). The number of binding interactions of individual POM with S100A9 is varied between one and four (Figure 5C,F). The interacting atoms are less than 3.5 Å apart, and the interaction angles are less than 25°. The rate of the interaction build-up is dependent on ionic strength. MD calculations at 20 mM NaCl lead to complex formation within 10 ns (Figure 5C,F), while MD calculations at 150 mM NaCl do not show complex formation within 150 ns (data not shown). Ionic strength affects the rates of complex formation due to the excluded volume effect.^56^ The ligand binding does not produce significant changes in the rest of the protein structure (Figure 5I,L), which remains unperturbed, apart from the intrinsically highly mobile N- and C-termini. RMSF graphs demonstrate that only the POM binding site (residues 50–55) and the Ca^2+^ binding EF-hand structural motifs (residues 25–30 and 62–70) are affected, which constitutes ca. 16% of all residues. Both ligands are highly mobile in the complex with average RMSD values of 5.2 and 6.7 for Nb_10_ and TiNb_9_, respectively (data not shown).

### POM Binding to Native S100A9 Monitored by Intrinsic Fluorescence

**Figure 6:**
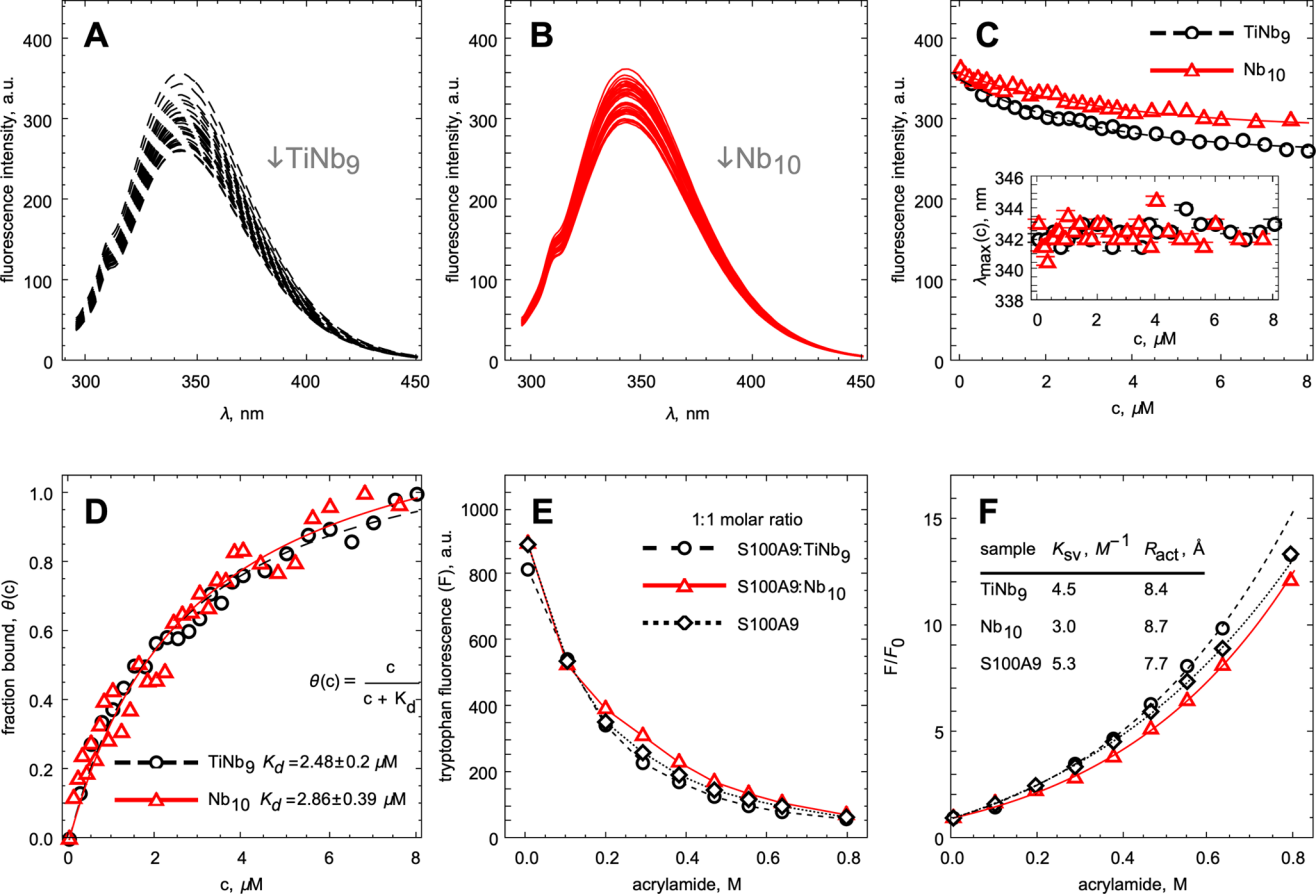
Binding of TiNb_9_ and Nb_10_ to native S100A9 monitored by intrinsic fluorescence. (A, B) S100A9 fluorescence emission spectra in the presence of increasing TiNb_9_ (shown in black) and Nb_10_ (shown in red) concentrations. (C) Maximum fluorescence intensity *versus* TiNb_9_ and Nb_10_ concentrations, respectively. Maxima of fluorescence spectra *versus* TiNb_9_ and Nb_10_ concentrations are shown in the inset. The lines are plotted only to guide the eye. (D) Titration plot of S100A9 by TiNb_9_ and Nb_10_, respectively, and corresponding fitting by one binding site model as indicated in the inset. (E) Maximum fluorescence intensity of native S100A9 alone and with the S100A9 to POMs molar ratios of 1:1 *versus* increasing concentrations of fluorescence quencher acrylamide. The lines are drawn only to guide the eye. The symbols in (E, F) are encoded as indicated in the inset in (E). (F) Stern–Volmer plots for the fluorescence quenching curves shown in (E). Lines in (F) indicate the fitting by the quenching sphere of action model. Stern–Volmer quenching constants and quenching sphere of action radii calculated for each sample are shown in the inset to (F). 4 μM S100A9, 50 mM HEPES, pH 7.0, and 20 °C.

Since S100A9 contains one tryptophan residue in its structure, Trp88, the binding of Nb_10_ or TiNb_9_ to the S100A9 native state was monitored by intrinsic fluorescence (Figure 6). The intensities of the fluorescence spectra of S100A9 decreased upon addition of either POM, while the spectral maxima did not change (Figure 6A,B). This indicates that POM binding induced the conformational changes in Trp88 surrounding, manifested in the reduction of tryptophan fluorescence intensity, but did not cause the significant perturbation of its dipolar moment, as the spectral maximum position was not affected. This suggests that there are no global changes in S100A9 conformation upon POM binding, which is consistent with the above results of MD simulation also indicating the lack of global structural perturbation in S100A9 homo-dimer upon POM binding. The fitting of the titration curves with a single binding site model yielded in the very close values of dissociation constants of *K*_d_ = 2.48 ± 0.2 μM for TiNb_9_ and *K*_d_ = 2.86 ± 0.39 μM for Nb_10_, respectively (Figure 6D). These values are consistent with the binding model found in MD simulation.

The intrinsic fluorescence intensities of S100A9 alone and in the complexes with POMs at 1:1 molar ratios were quenched by using noncharged fluorescence quencher acrylamide, as shown in Figure 6E. The quenching curves were transformed into the Stern–Volmer plots,^38^ as shown in Figure 6F. The Stern–Volmer plots for S100A9 alone and for the complexes with POMs were characterized by very similar dependences, which all deviate from linearity. The upward-curving of the Stern–Volmer plots could be due to the adjacence of quencher to fluorophore at the moment of excitation, and therefore, we used the quenching sphere of action model to analyze the acrylamide quenching data.^38^ All three plots were well described by the following Stern–Volmer constants and quenching sphere of action radii, respectively: 5.24 M^–1^ and 7.7 Å for S100A9 alone, 4.5 M^–1^ and 8.4 Å for the S100A9-TiNb_9_ complex, and 3.0 M^–1^ and 8.7 Å for the S100A9-Nb_10_ complex. These parameters indicate only slight changes in the Trp 88 environment upon POM binding to S100A9.

### POM Binding to Native and Amyloid S100A9 Monitored by ANS Fluorescence

**Figure 7:**
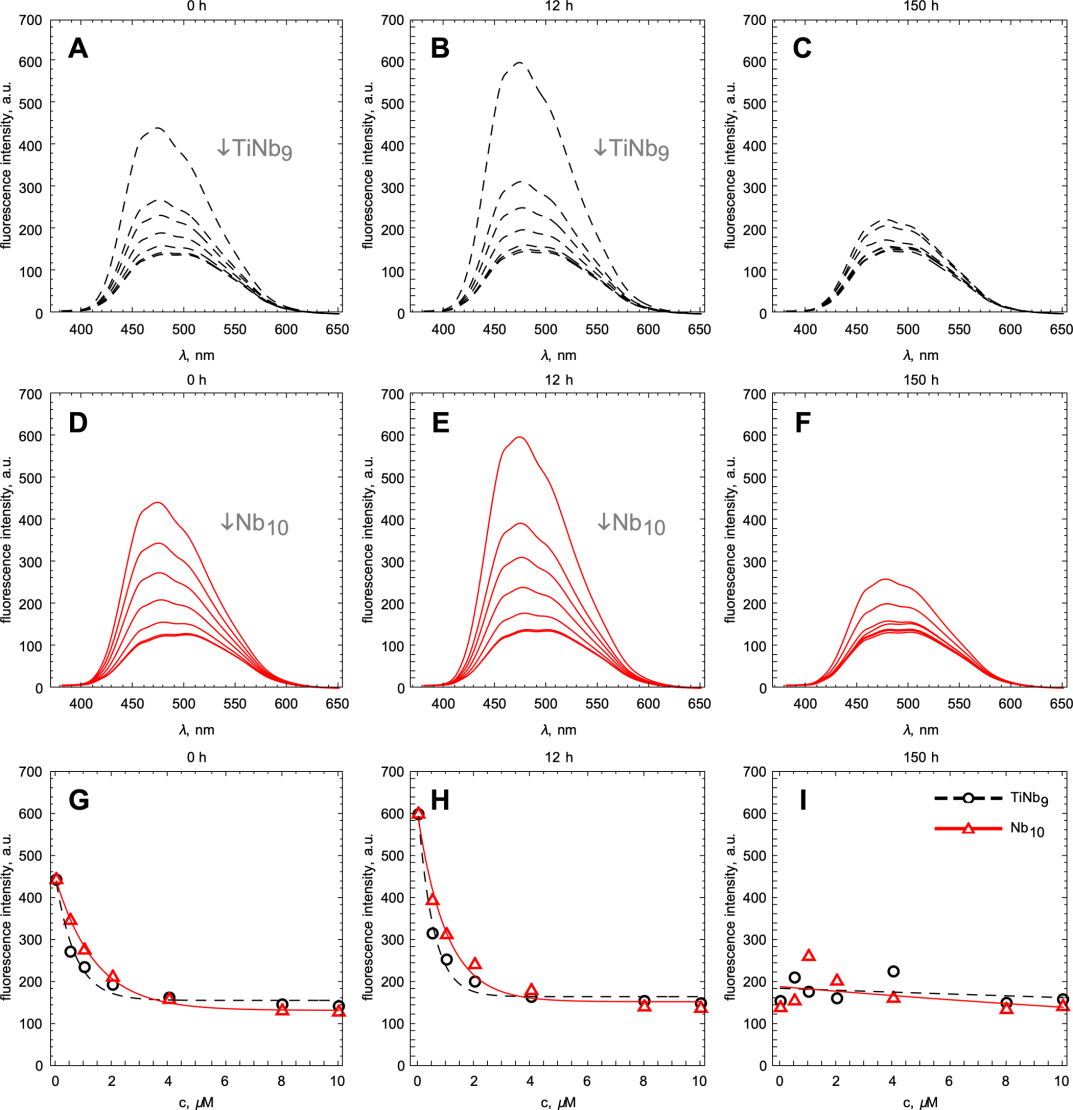
TiNb_9_ and Nb_10_ binding to native and amyloid S100A9 monitored by ANS fluorescence. (A, D) Changes in ANS fluorescence spectra upon titration of native S100A9 by TiNb_9_ (shown in black) and Nb_10_ (shown in red), respectively. (B, C, E, F) Changes in ANS fluorescence spectra of the amyloid complexes of S100A9 formed in the presence of increasing concentrations of TiNb_9_ and Nb_10_ after 12 and 150 h incubation, respectively. (G) Titration curves of native S100A9 with TiNb_9_ and Nb_10_ fitted by one binding site model. (H, I) Changes in ANS fluorescence intensities upon binding to the S100A9-POM complexes incubated for 12 and 150 h, respectively. An increase in TiNb_9_ and Nb_10_ concentrations is illustrated by arrows. The changes in ANS fluorescence intensities for the S100A9-Nb_10_ are shown by a red solid line and red symbols, and those for the S100A9-TiNb_9_ are shown by a black dashed line and black symbols. 15 μM S100A9 (monomer equivalent), 120 μM ANS, 50 mM Hepes, pH 7.0, and 42 °C.

Binding of TiNb_9_ and Nb_10_ to native and amyloid S100A9 was monitored also by ANS fluorescence since ANS dye binds to both hydrophobic and positively charged patches on protein surfaces.^58^ Interestingly, we have observed the decrease in ANS fluorescence upon titration of native S100A9 with either POM. This may reflect that POMs interact with the Lys-rich cluster on the S100A9 surface, identified above in MD simulation, and this makes this cluster inaccessible for ANS binding (Figure 7A,D). The fitting of the titration curves with a single-binding site model resulted in *K*_d_ values of 0.45 ± 0.03 μM for TiNb_9_ and 1.17 ± 0.03 μM for Nb_10_, respectively (Figure 7G), which are close to those determined by the intrinsic fluorescence experiments described above.

Interestingly, ANS binding increased after 12 h incubation of S100A9 alone, when intermediate amyloid species were self-assembled, indicating their potentially higher surface hydrophobicity compared to native S100A9 (Figure 7B,E). However, S100A9 incubated for 12 h in the presence of increasing POM concentrations is characterized by a reducing ANS signal, which become comparable to ANS bound to native S100A9-POM complexes at the respective POM concentrations. This indicates that in the intermediate S100A9 amyloid species, POMs still can bind to the Lys-rich cluster and therefore this cluster is not completely buried within their interior. When S100A9 alone was incubated for 150 h, we observed a substantial reduction of ANS binding compared to the native and partially fibrillated S100A9, reflected in the reduction of its fluorescence (Figure 7C,F,I). Previously, we have observed the clumping of S100A9 amyloid fibrils upon prolonged incubation,^32,54^ which may lead to a decrease in their accessible hydrophobic surfaces and therefore ANS binding. When S100A9 was incubated with POMs for 150 h, the fluorescence intensity of ANS bound to these complexes did not change significantly compared to S100A9 incubated alone, indicating that Lys-rich patches are potentially buried within their interior, while their hydrophobic surfaces are accessible to ANS.

### POMs as Inhibitors of S100A9 Amyloid Formation and Relevance to Neurodegenerative Diseases

S100A9 plays a critical role in the initiation and progression of neurodegenerative diseases such as Alzheimer’s, Parkinson’s, and traumatic brain injury, where brain injury is viewed as a precursor state for neurodegeneration.^22,23,29^ S100A9 is characterized by both pro-inflammatory properties and ability to undergo spontaneous amyloid self-assembly under the physiological conditions, being the central component of the amyloid-neuroinflammatory cascade.^22,23,29^ The inhibition of S100A9 amyloid formation is of significant interest since this may affect the whole cascade of events and mitigate the disease development. Here, we have demonstrated that increasing concentrations of both polyoxoanions TiNb_9_ and Nb_10_ can effectively inhibit the amyloid kinetics of S100A9 (Figure 2). The effect is very pronounced already at the 1:0.5 molar ratio of S100A9 to corresponding POM, and at the molar ratios of 1:1 and even more so at 1:10, the whole amyloid self-assembly is effectively abolished. Increasing concentrations of POMs both reduce effectively the rate of amyloid assembly and quantity of formed amyloids (Figure 2). The AFM imaging revealed that the long fibrillar structures of S100A9 are completely abolished at the elevated POM concentrations and instead round-shaped compact aggregates or very short elongated stretches are developed (Figure 3).

Despite significant efforts over the past decades, there are still no drugs available to avert amyloid neurodegenerative disease progression and the overall failure rate in the clinical trials is very high even for compounds that have shown promising anti-amyloid activity *in vitro*. Various inhibitors have been developed for arresting or modulating the amyloid aggregation, including peptides and peptidomimetics,^59,60^ nanoparticles,^61^ chaperons,^62^ and molecular tweezers.^63^ Among them, antibodies^64,65^ have received significant attention due to their high specificity toward their targets, despite their relatively high production cost. However, raising antibodies against such targets as amyloidogenic polypeptides and their epitopes, which may take part in various biological processes, is a challenging task and potent compounds without significant side effects on the complementary pathways are still needed for effective therapeutic treatments. Small molecules are preferred from a pharmacological perspective as they are of small size, high permeability, high efficiency toward their targets and their cost is relatively low.

POMs as important inorganic nanosized drug compounds based on early transition metal-oxygen-anion clusters were in the center of biomedical research for the past decades due to their anti-viral and anti-tumor activities.^11,66−71^ Recently, there was significant interest in the synthesis and applications of Nb- and Ta-based POMs, which are inert, do not undergo redox reactions in water, and are stable under neutral and alkaline conditions.^12,72,73^ In addition, the much higher charge of polyoxoniobates than Mo- and W-based POMs can be achieved since Nb has an oxidation state of +5. By replacing Nb with Ti in the POM, the charge of the molecule can be further increased. Since the interactions between POMs and biomolecules are of electrostatic nature,^72,73^ the increased POM charge may result in the increased specificity of these compounds. Thus, polyoxoniobates have been proven to be highly attractive candidates for biological applications, including the treatment of amyloid and neurodegenerative diseases. Moreover, oxide-covered Nb and Ta devices have shown high biocompatibility without known adverse effects in the orthopedic and dental implants.^74−77^

The W-based POMs and POM derivatives with a defined His chelating binding sites were identified as functional anti-amyloid agents in inhibiting Aβ peptide fibrillation in Alzheimer’s disease via electrostatic interactions.^10,78,79^ Importantly, POMs were demonstrated to be able to cross the blood–brain barrier^10^ and therefore they can reach the brain tissues, where the amyloid accumulation and the pathological changes take places in neurodegenerative diseases.

Thus, as TiNb_9_ and Nb_10_ have shown to inhibit S100A9 amyloid formation, the next question, which we have addressed here, is how POMs interact with native protein or with developing amyloid aggregates. It has been shown that in Aβ peptide, POMs bind to the cationic cluster His-His-Gln-Lys, driven by charge interactions and possibly His chelating effects.^10^ We have examined the prospective binding site of TiNb_9_ and Nb_10_ with native S100A9 homo-dimer by using the combination of methods including MD simulation, the direct titrations of native S100A9 with POMs monitored by intrinsic and ANS extrinsic fluorescence, acrylamide quenching of intrinsic fluorescence of S100A9 without and with bound POMs, and CD in the far and near UV regions. MD simulation illustrated that both Nb_10_ and TiNb_9_ can bind via dynamic ionic interactions to the very flexible region of S100A9 native homo-dimer, containing the cluster of Lys residues, i.e., Lys50, Lys51, and Lys54 and in addition involving either Lys106 from the same S100A9 monomer for TiNb_9_ or Lys4 from another S100A9 monomer for Nb_10_. Interestingly, this condensed positively charged cluster is involved in the region with high amyloid propensity as identified for the S100A9 amino acid sequence previously^30^ and located between two EF-hand calcium-binding motifs in each S100A9 monomer. The titration experiments monitored by intrinsic fluorescence revealed that both POMs bind to native S100A9 with a *K*_d_ of ca. 2.5 μM. The similar binding affinity of POMs to S100A9 was confirmed upon titration monitored by ANS fluorescence. The acrylamide quenching of Trp 88 in the native S100A9 and S100A9 in complexes with POMs demonstrated that its environment did not change upon POM binding. The far and near UV CD also showed that the secondary and tertiary structure of S100A9 were not perturbed on the large scale detectable by CD upon POM binding. All these data indicate that POMs most likely induce only local conformational changes in the binding sites, rather than the global perturbation of protein molecule and overall stabilization of protein structure.

Interestingly, while POMs inhibit the amyloid assembly of S100A9, when added in the beginning of the process, they did not produce any significant effect, when added to preformed S100A9 amyloids as monitored by ThT binding and AFM imaging (Figure 3).

Thus, all data from the MD simulation and fluorescence titration experiments suggests that Lys-rich patches are involved in S100A9 amyloid formation and blocking them by POMs in the beginning of the process will abolish the amyloid formation *per se*. If these patches are already involved in the S100A9 amyloid formation, they became inaccessible to POMs and POMs do not produce disaggregating effect on preformed S100A9 amyloids. Therefore, we have identified a very targeted mechanism to inhibit S100A9 amyloid formation and via S100A9 affect the whole amyloid-neuroinflammatory cascade.

## Conclusions

By using the ThT fluorescence kinetics experiments and AFM imaging, we have demonstrated that both Nb_10_ and TiNb_9_ can effectively inhibit S100A9 amyloid formation, completely abolishing the growth of long filamentous structures observed in the absence of POMs. Combining MD and fluorescence titration experiments, we have demonstrated that both POMs interact with positively charged Lys-rich patches on the native S100A9 surface, which are most likely the key sequences involved in S1009 amyloid self-assembly. The inhibition and complete hindering of S100A9 amyloid pathways may be used in therapeutic applications targeting the amyloid-neuroinflammatory cascade in the neurodegenerative diseases.

## Supporting Information Available

APBS calculations show that S100A9 homo-dimer induces the negative electric potential in the surrounding space with the distinct areas of positive electric field (PDF)

## ABBREVIATIONS

AFM: atomic force microscopy
ANS: 8-anilino-1-naphthalene sulfonate
CD: circular dichroism
MD: molecular dynamics
POM: polyoxometalate
RMSD: root-mean-square displacement
RMSF: root-mean-square fluctuations
ThT: thioflavin-T
