# Supplementary material for: Polyoxometalates as Effective Nano-inhibitors of Amyloid Aggregation of Pro-inflammatory S100A9 Protein Involved in Neurodegenerative Diseases
Source: ACS Appl Mater Interfaces. 2021 Jun 3;13(23):26721–34. doi: 10.1021/acsami.1c04163 (PMC8289188; doi:10.1021/acsami.1c04163)
Supplement: Supplementary file 1 — am1c04163_si_001.pdf [file am1c04163_si_001.pdf]

Supporting Information

Polyoxometalates as effective nano-inhibitors of amyloid aggregation of pro-inflammatory S100A9 protein involved in neurodegenerative diseases.

Himanshu Chaudhary,[‡] *Igor A. Iashchishyn,*[‡] *Nina V. Romanova, Mark A. Rambaran, Greta Musteikyte, Vytautas Smirnovas, Michael Holmboe, C. André Ohlin,*[*] *Željko M. Svedružić,* [*] *Ludmilla A. Morozova-Roche* [*]

KEY WORDS: Amyloid, amyloid-neuroinflammatory cascade, fibrils, inhibition, S100A9, polyoxometalate, decaniobate, titanoniobate.

Corresponding Authors

Ludmilla A. Morozova-Roche – *Department of Medical Biochemistry and Biophysics, Umeå University, 90187 Umeå, Sweden;* ORCHID: 0000-0001-5886-2023;

Phone +46736205283, +46907865283; Email: ludmilla.morozova-roche@umu.se

Željko M. Svedružić – *Department of Biotechnology, University of Rijeka, HR 51000 Rijeka, Croatia;* ORCHID 0000-0002-0736-6182; Email: zeljko.svedruzic@biotech.uniri.hr

André Ohlin – *Department of Chemistry, Umeå University, 90187 Umeå, Sweden;*

ORCHID 0000-0002-3804-6421; Email: andre.ohlin@umu.se

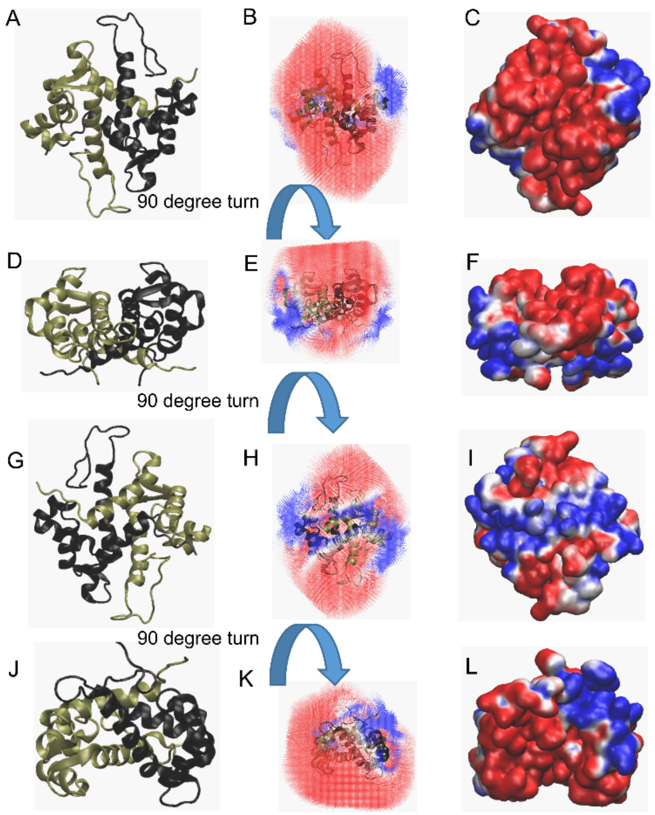

Figure S1. APBS calculations show that S100A9 homo-dimer induces the negative electric potential in the surrounding space with the distinct areas of positive electric field. (A, D, G, J) The ribbon diagrams in different projections demonstrating homo-dimer of S100A9, in which one monomer is shown in lighter and another – in darker color. (B, E, H, K) Isopotential lines in the space around S100A9 homo-dimer are shown in red-white-blue and correspond to -0.5, 0, +0.5 kB*T/e. (C, F, I, L) Potentials on the Connolly surface of S100A9 homo-dimer are shown in red-white-blue and correspond to -2, 0, 2 kB*T/e. Each figure corresponds to 90 degree rotation as shown by arrows between subsequent images.
